# Strong, spectrally-tunable chirality in diffractive metasurfaces

**DOI:** 10.1038/srep13034

**Published:** 2015-09-04

**Authors:** Israel De Leon, Matthew J. Horton, Sebastian A. Schulz, Jeremy Upham, Peter Banzer, Robert W. Boyd

**Affiliations:** 1 Department of Physics and Max Planck Centre for Extreme and Quantum Photonics, University of Ottawa, 25 Templeton, Ottawa, ON, K1N 6N5, Canada; 2 Institute of Optics and Department of Physics and Astronomy, University of Rochester, Rochester NY, 14627.

## Abstract

Metamaterials and metasurfaces provide a paradigm-changing approach for manipulating light. Their potential has been evinced by recent demonstrations of chiral responses much greater than those of natural materials. Here, we demonstrate theoretically and experimentally that the extrinsic chiral response of a metasurface can be dramatically enhanced by near-field diffraction effects. At the core of this phenomenon are lattice plasmon modes that respond selectively to the illumination’s polarization handedness. The metasurface exhibits sharp features in its circular dichroism spectra, which are tunable over a broad bandwidth by changing the illumination angle over a few degrees. Using this property, we demonstrate an ultra-thin circular-polarization sensitive spectral filter with a linewidth of ~10 nm, which can be dynamically tuned over a spectral range of 200 nm. Chiral diffractive metasurfaces, such as the one proposed here, open exciting possibilities for ultra-thin photonic devices with tunable, spin-controlled functionality.

The recent progress in nanoscience and nanofabrication technology has enabled the development of artificial materials, whose optical properties can be controlled by design^1^,^2^,^3^. Such metamaterials typically consist of metallic nanoparticles that support surface plasmon resonances determined by their morphology and material composition^4^. Recently, it has been recognized that two-dimensional metamaterials, or metasurfaces, hold great potential for manipulating the properties of light using planar geometries, offering a route for developing ultra-thin optical devices with sophisticated functionalities^5^,^6^,^7^,^8^,^9^,^10^. Within this context, the study of chiral metasurfaces has attracted much attention. Chiral metasurfaces are composed of nanoparticles that lack in-plane mirror symmetry. They possess an intrinsic sense of handedness that allows them to interact differently with right and left circularly polarized light. Consequently, they can exhibit chiral optical effects, such as circular polarization conversion, asymmetric transmission of circularly polarized light, and extraordinary circular dichroism (CD) and optical rotation^11^,^12^,^13^,^14^,^15^,^16^,^17^. Furthermore, the strength of some of these phenomena can be dynamically controlled in metasurfaces operating at terahertz frequencies^18^,^19^. Optical chirality can also be observed in achiral metasurfaces by breaking the mirror symmetry of the entire experimental arrangement through the use of oblique illumination^20^,^21^,^22^,^23^,^24^,^25^,^26^,^27^,^28^. This phenomenon is known as extrinsic chirality. In such a system, the strength of the chiral response can be controlled by the degree of asymmetry introduced by the oblique illumination, while the handedness of the system is given by the sign of the angle of incidence. Such characteristics make these systems extremely important for the development of dynamically controlled polarization spectral filters and optical rotators^26^,^27^,^28^.

Despite the great flexibility for designing chiral metamaterials and metasurfaces, a major challenge in the field has been the development of structures with a chiral response that is dynamically tunable over a broad spectral range. This challenge arises because the spectral properties of such structures depend primarily on the nanoparticle dimensions and shape, which cannot be adjusted dynamically. The lack of dynamic spectral tunability limits the prospects for applications and tightens the fabrication requirements. To overcome this problem, alternative approaches for metasurfaces with a strong and spectrally-tunable chiral response must be developed.

Here we report a new approach for chiral metasurfaces, which results in the first demonstration of a chiral structure with a broad, continuous spectral tunability. This approach is based on a fundamentally different manifestation of extrinsic chirality, where near-field diffraction effects play a key role. In our case, the strength of the chiral response is not imposed by the degree of asymmetry introduced by the oblique illumination, but rather by the diffractive properties of the metasurface. At the centre of this phenomenon is the excitation of lattice surface modes (LSMs)–delocalized plasmon-photon modes enabled by diffractive coupling of localized plasmons^29^,^30^,^31^,^32^,^33^–that respond selectively to the handedness of the incident circularly polarized light. We shall refer to these modes as chiral LSMs. The rich dispersive properties of these LSMs offer a way to control dynamically the spectral response of the system. Contrary to the extrinsic chirality observed in previous investigations^21^,^22^,^23^,^24^,^25^,^26^,^27^,^28^, the diffraction-assisted phenomenon reported here enables an extraordinary CD for illumination close to normal incidence, and exhibits a spectral response that is extremely sensitive to the illumination angle. We demonstrate experimentally that the proposed metasurface can be used as an ultra-thin circular-polarization spectral filter at near infrared wavelengths, achieving a tuning range of 200 nm with a linewidth of ~10 nm by simply varying the illumination angle over a range of only 16.5°.

## Results

### Optical modes of the metasurface

**Figure 1:**
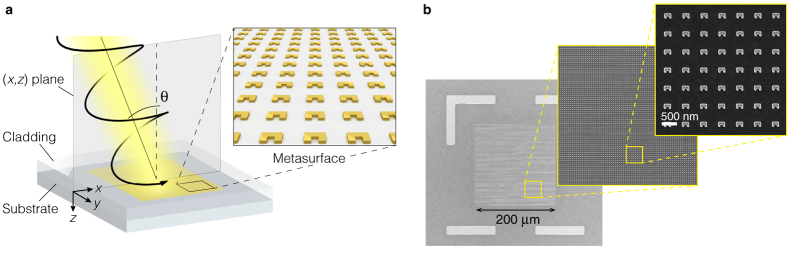
Metasurface under investigation. (**a**) Schematic illustration of the metasurface and illumination configuration. The metasurface consists of gold SRRs arranged in a square lattice geometry. The SRR array is supported by a glass substrate, and cladded with a medium index-matched to the substrate. The structure is illuminated by circularly polarized white light, propagating in the (*x*, *z*) plane and making an angle *θ* with respect to the metasurface normal. (**b**) Scanning electron microscope images of the fabricated structure.

The metasurface consists of an array of gold split ring resonators (SRRs) arranged in a square lattice geometry, supported by a glass substrate, and cladded by a medium that is index matched to the substrate (see Fig. 1a). The lattice spacing of the metasurface, Λ = 600 nm, is made deliberately large compared to the size of the SRRs in order to allow diffraction effects. The metasurface extends over the (*x*, *y*) plane with the symmetry axis of the SRRs oriented along the *y* direction. The structure is illuminated from the cladding side by circularly polarized light that propagates in the (*x*, *z*) plane making an angle *θ* with respect to the metasurface normal. Figure 1b shows various scanning electron microscope images of the fabricated structure. The SRRs have a square U shape with outer dimensions of 240 nm by 165 nm and a thickness of ~80 nm.

To understand the diffraction-assisted extrinsic chiral response of the metasurface, we must consider the various resonances of a single SRR and how they interact in a lattice. For this task, it is instructive to decompose the incident circularly polarized electric field into *s*- and *p*-polarizations, i.e., **E**_s_ = [0, *E*_*y*_, 0] and **E**_p_ = [*E*_*x*_, 0, *E*_*z*_], as we can directly relate the resonances of a single SRR to this polarization basis.

**Figure 2:**
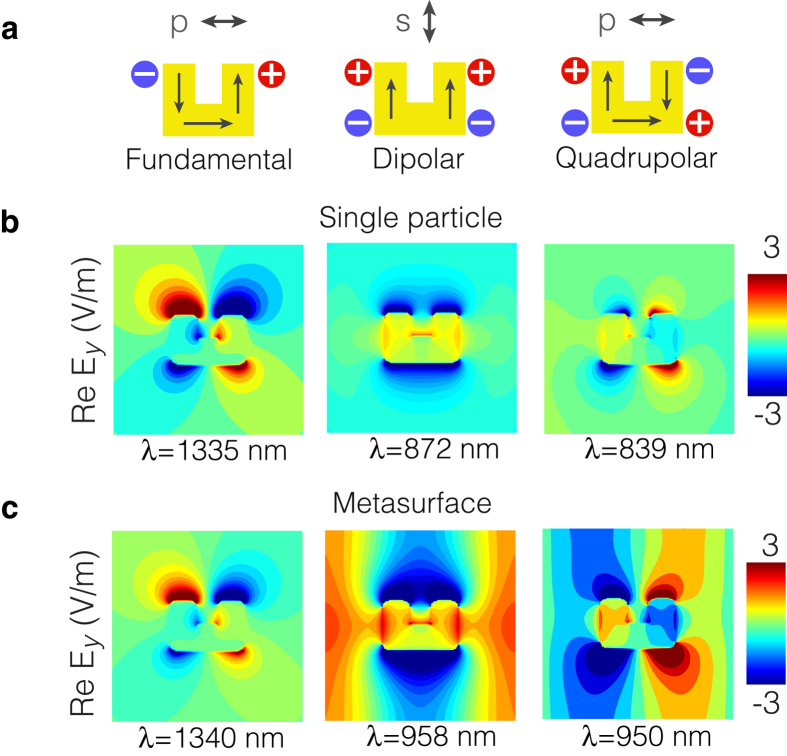
Optical modes of the single SRR and of the metasurface. (**a**) Schematic representation of the surface current density (arrows) and charge distributions characterizing the three main resonances of a single SRR. The polarization state required to excite these resonances is indicated by the arrows above. (**b**) Real part of the *E*_*y*_ near-field distribution corresponding to the resonance indicated directly above in **a** for the case of a single SRR. (**c**) Same as in **b** but for the **SRR array** (plotted over one unit cell). The wavelength associated with each resonance is indicated under the corresponding field distribution.

The three main resonances of a single SRR are characterized by the surface current and charge distributions depicted schematically in Fig. 2a. The fundamental resonance is excited by *p*-polarized light as the electric field induces an electric dipole moment across the gap region and a surface current that flows along the SRR^34^. Here, our main focus is not on this resonance, but rather on the two next higher-order resonances, which are dipolar and quadrupolar in nature. The dipolar resonance is excited by *s*-polarized light because the electric field induces electric dipole moments and current distributions that oscillate in phase along the vertical arms of the SRR, while the quadrupolar resonance is excited by *p*-polarized light because the electric field induces an electric dipole moment along the horizontal arm of the SRR^35^. The electromagnetic fields associated with these resonances are obtained employing a Maxwell’s equations solver based on the finite-difference time-domain method, assuming normal incidence excitation (see Methods). The calculated real part of the *E*_*y*_ near-field distributions are shown in Fig. 2b, with the peak resonance wavelengths indicated accordingly.

When these nanoparticles are arranged in a lattice with a spacing comparable to the optical wavelength, the resonances of the individual particles can couple strongly with those of neighbouring particles via grazing diffraction orders, or Rayleigh anomalies (RAs). This results in the formation of LSMs at wavelengths close to the RA condition, given by^31^.


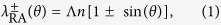


where *n* is the refractive index of the medium surrounding the nanoparticles and only the first positive (+) and negative (−) diffraction orders have been considered. For the metasurface at hand and under normal incidence, a RA occurs at *λ*_RA_ = 905 nm, which is close to the excitation wavelengths of the dipolar and quadrupolar resonances of the single SRR. The coupling of these high-order particle resonances via the RA results in LSMs that preserve the field symmetries of the particle’s mode. This is illustrated Fig. 2c, which shows the *E*_*y*_ near-field distributions of the modes of the metasurface plotted over one unit cell. A LSM is not formed at the fundamental resonance because it occurs far from the RA condition. Consequently, the optical mode of the metasurface at this resonance is practically unchanged.

For both dipolar and quadrupolar LSMs, *E*_*y*_ is the dominant electric field component, and at normal incidence they form standing waves due to an equal amounts of light diffracted in the ± *x* directions. The quadrupolar LSM also has a non-zero *E*_*x*_ field component; however it is much smaller than *E*_*y*_, and will be neglected in the following analysis.

### Oblique excitation of mixed (dipolar-quadrupolar) LSMs

**Figure 3:**
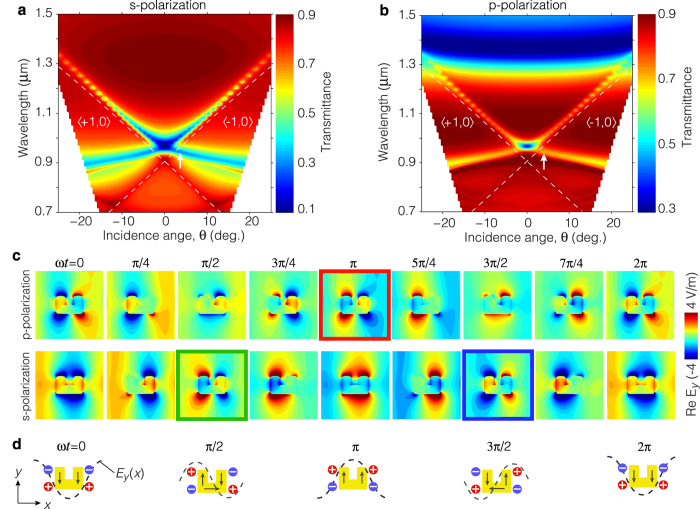
Excitation of mixed (dipolar-quadrupolar) LSMs using oblique illumination. (**a**,**b**) Transmittance spectra of the metasurface calculated for *s*- and *p*-polarized light as functions of the illumination angle, *θ*. The spectral features that follow the Rayleigh anomaly condition (white dashed lines) denote the excitation of LSMs. (**c**) Time evolution of the forward propagating LSMs excited with *s*- and *p*-polarized light at *θ* = 3.5° and *λ* = 935 nm (indicated by the arrows in **a** and **b**). The fields of these LSMs are dephased by *π*/2 radians with respect to each other and their time evolution reveals a dynamic interplay between the dipolar and quadrupolar resonances. (**d**) Schematic description of the excitation of the dipolar and quadrupolar resonances of a single SRR by a planewave. The dashed curves represent the spatial electric field distribution of a *y*-polarized planewave propagating along the +*x* direction. The excitation of the dipolar and quadrupolar resonances by the planewave alternates every quarter of an optical cycle.

We now consider the case of oblique incidence illumination. The transmittance spectra calculated for *s*- and *p*-polarized illumination are plotted as functions of the incidence angle in Fig. 3a,b, respectively. The dashed lines superimposed on the figure indicate the conditions for the 〈± 1, 0〉 RA, given by Eq. (1). The various spectral features that follow these dashed lines correspond to the excitation of LSMs, while the angle-independent broad spectral feature observed at longer wavelengths (~1.4 *μ*m) in Fig. 3b corresponds to the fundamental resonance of the SRR. Critically, for oblique illumination the 〈± 1, 0〉 RA occur at different wavelengths and consequently, the dispersion characteristics of the LSMs develop into two branches, each of them corresponding to one of the RAs. Furthermore, under oblique illumination, conservation of momentum parallel to the metasurface leads to LSMs with well defined directions. Therefore, for each angle of incidence *θ *≠ 0 in Fig. 3a,b there exist two LSMs, one propagating in the forward direction (+*x*) associated with the 〈+1, 0〉 RA, and the other propagating in the backward direction (−*x*) associated with the 〈−1, 0〉 RA. Note that the spectral features associated with LSMs appear to be discontinuous for wavelengths around 1.2 *μ*m. This is simply a numerical artifact that arises due to the incidence angle resolution chosen for the simulations.

Consider for instance the forward-propagating LSMs excited using *p*- and *s*-polarized light at *θ* = 3.5° (marked by arrows in Fig. 3a,b). The top and bottom panels of Fig. 3c illustrate the calculated real part of the *E*_*y*_ near-field distributions of these LSMs as they evolve in time over the interval of one optical cycle [exp(*iωt*) time-harmonic dependence implicit]. Note that these modes cannot be ascribed to a single type of particle resonance (dipolar or quadrupolar), as for the case of normal illumination. Rather, the evolution of these fields reveals a dynamic interplay between the dipolar and quadrupolar resonances of the SRR. This phenomenon can be understood heuristically by considering the interaction between a single SRR and a *y*-polarized planewave propagating in the *x* direction, as shown schematically in Fig. 3d. Here, when *ωt* = 0 the electric field of the planewave (represented by the dashed curve) reaches its minimum at the central location of SRR, and therefore it induces electric dipole moments along the vertical arms that oscillate in phase (dipolar resonance). After a quarter of an optical cycle, the electric field vanishes at the central location of the SRR but exhibits equal amplitudes with opposite polarities at the locations of the vertical arms. In this case, the electric field induces electric dipole moments along the vertical arms that oscillate *π* radians out of phase, leading to an efficient excitation of the quadrupolar resonance. A similar process repeats over the second half of the optical cycle. By applying this reasoning to the modes of the metasurface, it is apparent that oblique illumination leads to propagating LSMs that exhibit both a dipolar and a quadrupolar character.

### Diffraction-assisted extrinsic chirality

The near-field distributions of the two LSMs depicted in Fig. 3c are similar to each other, yet they are dephased by a quarter of an optical cycle; i.e., the mode excited with *s*-polarized light lags the other by *ωt* = *π*/2. Thus, by exciting these LSMs simultaneously using **E**_p_ and **E**_s_ with an appropriate relative phase, one can exploit the near-field interference of these modes to manipulate the optical response of the metasurface. In particular, since these LSMs are dephased by a quarter of an optical cycle, they can undergo destructive or constructive interference when the metasurface is illuminated with circularly polarized light. This is, for a left-circularly polarized (LCP, **E**_L_ = **E**_p_−*i***E**_s_) excitation, constructive interference occurs due to the additional relative phase of *π*/2 between the two LSMs (the red and the green frames in Fig. 3c overlap temporally). On the other hand, for right-circularly polarized (RCP, **E**_R_ = **E**_p_+*i***E**_s_) excitation, destructive interference occurs due to the additional relative phase of −*π*/2 between the two LSMs (the red and the blue frames in Fig. 3c overlap temporally). Furthermore, since LCP and RCP light possess mirror symmetry with respect to each other, changing the sign of *θ* leads to constructive (destructive) interference for RCP (LCP) light.

**Figure 4:**
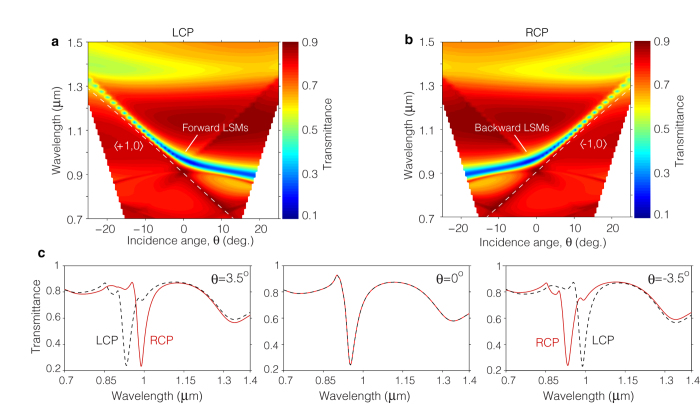
Chiral LSMs and diffraction-assisted extrinsic chirality. (**a**,**b**) Transmittance spectra of the metasurface calculated for circularly polarized light as functions of the illumination angle, *θ*. The spectral features that follow the Rayleigh anomaly conditions (white dashed lines) denote the excitation of chiral LSMs, which respond selectively to the handedness of circularly polarized light. (**c**) Simulated transmittance spectrum of the metasurface using circularly polarized illumination at three different angles of incidence. The excitation of chiral LSMs leads to a much greater extrinsic chiral response than that of the SRR’s fundamental resonance.

The metasurface exhibits a strong extrinsic chiral response that results from the near-field interference of these LSMs. This is evident from the transmittance spectra calculated for circularly polarized illumination (Fig. 4a,b), which exhibits mirror symmetry for opposite polarization handedness. This phenomenon is fundamentally different from the extrinsic chirality observed in other metasurfaces previously studied^21^,^22^,^23^,^24^,^25^,^26^,^27^,^28^. These earlier investigations exploit the bi-anisotropic response of the SRR’s fundamental resonance and as such, depend strongly upon the degree of asymmetry introduced by the oblique illumination. Consequently, a largely oblique illumination (*θ* > 30°) is required to observe significant chiral optical effects. In our case, the strength of the chiral response is not defined entirely by the external illumination, rather it is defined to a large extent by the diffractive properties of the metasurface. This distinct characteristic allows the metasurface to exhibit an extraordinary chiral response even for illumination angles as small as a few degrees. To exemplify this point we consider Fig. 4c, which shows the transmittance spectrum of the metasurface calculated using circularly polarized light incident at three different angles, *θ* = −3.5°, 0°, 3.5°. For a given polarization handedness, the spectrum exhibits two absorption features, one located around *λ* = 900 nm associated with the excitation of LSMs, and one located at *λ* = 1.35 *μ*m associated with the SRR’s fundamental resonance. The strength of the chiral response is characterized by the extent of dissimilarity in the transmittance spectra for RCP and LCP illumination. Note that dissimilar spectra occur only for *θ* ≠ 0, as expected for a system exhibiting extrinsic chirality. Clearly, the spectral dissimilarity at the SRR’s fundamental resonance is minute for the small angles of incidence considered. By comparison, the spectral features associated with the excitation of LSMs clearly present a much greater change upon changing the polarization handedness, indicating a much stronger extrinsic chiral response.

### Experimental observation of spectrally tunable extrinsic chirality

**Figure 5:**
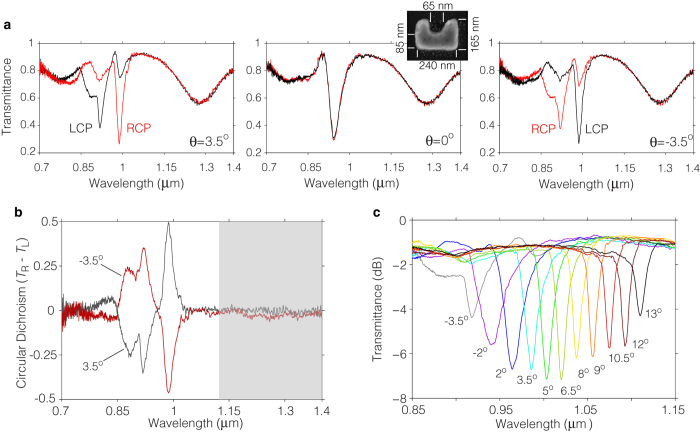
Experimental characterization of the metasurface. (**a**) Measured transmittance spectrum of the metasurface using circularly polarized illumination at the three angles of incidence simulated in Fig. 4c. The inset shows a typical scanning electron microscope image of one of the SRRs composing the metasurface. The results are in good agreement with the full wave simulations, and reproduce all the spectral features expected due to the excitation of chiral LSMs. (**b**) Measured CD spectra for *θ* = ± 3.5°, expressed as the differential transmittance using LCP and RCP illumination. The extrinsic chiral response of the metasurface is enhanced by the excitation of chiral LSMs (unshaded area). The measured CD in the shaded area is due to the extrinsic chiral response of the SRR’s fundamental resonance, which is not assisted by diffraction processes. (**c**) Measured transmittance using RCP illumination for various angles of incidence. The metasurface can be used as tunable polarization spectral filter with broad tuning range of 200 nm, achieved by varying the angle of incidence over only 16.5°. The spectral linewidth can be as narrow as 10 nm.

We fabricated a metasurface matching the design specifications discussed so far by employing standard nanofabrication techniques (see Methods). A thin layer of indium-tin oxide (ITO) exists between the glass substrate and the SRRs, enabling the gold to adhere to the substrate. This layer has been accounted for in the above simulations. The inset in Fig. 5a shows a typical scanning electron microscope image of one of the SRRs composing the metasurface, indicating its relevant dimensions. The thickness of the particle was measured to be 78 ± 1.7 nm.

In order to verify the theoretical predictions, we characterized experimentally the optical response of the metasurface via optical transmission spectroscopy (see Methods). Figure 5a shows the transmittance spectrum of the metasurface measured using circularly polarized illumination at the same three angles of incidence used for the calculations in Fig. 4c. The experimental results show very good agreement with the full wave simulations, and reproduce all the spectral features expected due to the excitation of chiral LSMs. The slightly different shape and shallower depth of the short wavelength transmittance dip can be attributed to fabrication inaccuracies such as a small asymmetry observed in the fabricated SRRs. We also note that the fundamental resonance of the SRR is blue shifted with respect to the theoretical prediction. This discrepancy is attributed to the effect of the ITO layer on the particle’s resonance^36^, which may not be accurately described by the complex permittivity of the ITO layer used in our simulations.

Figure 5b illustrates the CD spectrum of the metasurface at *θ* = ±3.5°, expressed as the difference in transmittance using RCP and LCP illumination. The peaks observed in the unshaded part of the spectrum are due to the diffraction-assisted chiral response of the metasurface (i.e., excitation of LSMs). On the other hand, the data in the shaded region is due to the extrinsic chiral response of the SRR’s fundamental resonance^21^,^23^. Clearly, the CD at the fundamental resonance is too weak to be distinguishable from the noise of our measurement system. In contrast, the diffraction-assisted CD is quite strong and amounts at its maximum to a 50% change in transmission between LCP and RCP. It is difficult to make an accurate assessment of the CD enhancement offered by the diffraction-assisted process, as the CD measurement at the fundamental resonance is dominated by noise. However, according to the simulated data, the enhancement is approximately 30-fold for this particular case.

The rich dispersive properties of the chiral LSMs supported by this metasurface enable us to tune dynamically its CD response over a broad spectral region spanning ~200 nm (see Fig. 4a,b). This great tunability is a key feature for the development of tuneable circular-polarization spectral filters. In Fig. 5c we show the performance of such a filter by measuring the transmittance spectra of the metasurface using RCP illumination at various angles of incidence in the range −3.5° < *θ *< 13°. Note that as the angle of incidence is varied, the absorption feature in these spectra shifts over a broad range spanning 200 nm with at least ~3 dB extinction. Furthermore, the spectral linewidth of this filter is remarkably narrow, ranging between 27 nm and 10 nm across the entire tuning range. These sharp linewidths enable us to fit up to eleven well separated filter channels over spectral tuning range of the filter. This device can be easily transformed into a tunable spectral filter for LCP light simply by flipping the sign of the incidence angles. Similar polarization spectral filters have been recently proposed^27^,^28^,^37^, however, these devices exhibit broad linewidths (>100 nm) and cannot offer a dynamic spectral tunability.

## Discussion and Conclusion

Our experimental results demonstrate that extrinsic chirality in metasurfaces can be dramatically enhanced by near-field diffraction effects. For the metasurface at hand, this occurs because high-order resonances of the individual SRRs hybridize with grazing diffraction orders leading to the formation of LSMs that selectively respond to the handedness of circularly polarized light. The rich dispersive properties of such chiral LSMs enable us to dynamically tune the chiral spectral response over a broad wavelength range, simply by varying the illumination angle over a few degrees. We also provide an intuitive understanding of the physics underlying the formation of chiral LSMs. Understanding how the chirality of such modes can be designed opens the door to develop tunable devices exhibiting other chiral optical phenomena, such as optical rotation, which are expected to occur in metasurfaces exhibiting diffraction-assisted extrinsic chirality. Interesting dispersive chiral phenomena, such as optical rotatory dispersion and polarization-handedness selective temporal dispersion, could also arise from the sharp resonances associated with the LSMs supported by this type of metasurfaces. Furthermore, because of its broad spectral tunability, diffraction-assisted extrinsic chirality holds potential for applications such as spin-selective optical wavelength multiplexing, as well as polarization controlled ultra-thin optical devices with sophisticated functionality, such as tuneable narrow-band polarization spectral filters and ultra-thin circular-polarization beam splitters.

## Methods

### Fabrication

The metasurface was fabricated by standard lift-off and electron beam (e-beam) lithography techniques, using a bilayer PMMA resist. The substrate consists of float glass with a 23 nm thick ITO layer. The ITO layer ensures charge dissipation during e-beam lithography and improves the adhesion of gold to the substrate. After pattern definition, a 78 ± 1.7 nm gold layer was deposited by thermal evaporation, followed by resist lift-off. A refractive-index matching fluid was dispensed on the metasurface in order to create an optically homogeneous environment around the nanoparticles. Subsequently, a thin coverslip was used to confine the fluid to the sample via surface tension.

### Numerical modelling

The plasmonic metasurface was modelled using a Maxwell’s equation solver based on the finite difference time domain method (Lumerical FDTD). The metasurface is assumed to be infinitely large in the plane of periodicity by applying Bloch boundary conditions to the boundary of the unit cell. The dispersion of the materials composing the metasurface was taken into account by using their frequency-dependent permittivities. The permittivities of glass and gold were obtained from the Refs. 38 and 39, respectively, while the permittivity of the ITO layer was obtained from spectroscopic ellipsometry measurement of a similar substrate sample.

### Experiments

Oblique incidence transmittance spectroscopy measurement were performed using a nearly collimated light beam generated by a stabilized tungsten-halogen lamp. The light spectrum covers the range 0.7 *μ*m < *λ *<1.4 *μ*m. The spectrally broad light beam was circularly polarized using a broadband linear polarizer followed by an achromatic quarter waveplate with its fast axis oriented at  ± 45° with respect to the polarizer’s axis. The circularly polarized beam was then weakly focused to a spot size of ~200 *μ*m so that the entire beam fits in the area defined by the metasurface. Subsequently, the transmitted light passes through an iris that filters large angular components. The beam after the iris (divergence half-angle < 1.5°) was coupled into a multimode optical fibre and its intensity was measured using an optical spectrum analyzer (Yokogawa AQ6370C).

## Additional Information

**How to cite this article**: De Leon, I. *et al.* Strong, spectrally-tunable chirality in diffractive metasurfaces. *Sci. Rep.* **5**, 13034; doi: 10.1038/srep13034 (2015).
